# Large mismatch profile predicts rapidly progressing brain edema in acute anterior circulation large vessel occlusion patients undergoing endovascular thrombectomy

**DOI:** 10.3389/fneur.2022.982911

**Published:** 2023-01-04

**Authors:** Yanqi Shao, Xinyi Chen, Huiyuan Wang, Yafei Shang, Jie Xu, Jinshi Zhang, Peng Wang, Yu Geng

**Affiliations:** ^1^Department of Neurology, Center for Rehabilitation Medicine, Zhejiang Provincial People's Hospital, Affiliated People's Hospital, Hangzhou Medical College, Hangzhou, Zhejiang, China; ^2^Second Clinical Medical College, Zhejiang Chinese Medical University, Hangzhou, China; ^3^Department of Clinical Medicine, Bengbu Medical College, Bengbu, China; ^4^Department of Nephrology, Urology and Nephrology Center, Zhejiang Provincial People's Hospital, Affiliated People's Hospital, Hangzhou Medical College, Hangzhou, Zhejiang, China

**Keywords:** brain edema, acute ischemic stroke, endovascular thrombectomy, CT-perfusion, ischemic core, penumbra, mismatch ratio

## Abstract

**Background:**

Brain edema is a severe complication in patients with large vessel occlusion (LVO) that can reduce the effectiveness of endovascular therapy (EVT). This study aimed to investigate the association of the perfusion profile at baseline computed tomography (CT) perfusion with rapidly progressing brain edema (RPBE) after EVT in patients with acute anterior LVO.

**Methods:**

We retrospectively reviewed consecutive data collected from 149 patients with anterior LVO who underwent EVT at our center. Brain edema was measured by the swelling score (0–6 score), and RPBE was defined as the swelling score increased by more than 2 scores within 24 h after EVT. We investigated the effect of RPBE on poor outcomes [National Institute of Health Stroke Scale (NIHSS) score and modified Rankin scale (mRS) score at discharge, the occurrence of hemorrhagic transformation, and mortality rate in the hospital] using the Mann–Whitney *U*-test and chi-square test. A multivariate logistic regression model was used to assess the relationship between perfusion imaging parameters and RPBE occurrence.

**Results:**

Overall, 39 patients (26.2%) experienced RPBE after EVT. At discharge, RPBE was associated with higher NIHSS scores (*Z* = 3.52, 95% CI 2.0–12.0, *P* < 0.001) and higher mRS scores (*Z* = 3.67, 95% CI 0.0–1.0, *P* < 0.001) including the more frequent occurrence of hemorrhagic transformation (χ^2^ = 22.17, 95% CI 0.29–0.59, *P* < 0.001) and higher mortality rates in hospital (χ^2^ = 9.54, 95% CI 0.06–0.36, *P* = 0.002). Univariate analysis showed that intravenous thrombolysis, baseline ischemic core volume, and baseline mismatch ratio correlated with RPBE (all *P* < 0.05). After dividing the mismatch ratio into quartiles and performing a chi-square test between quartiles, we found that the occurrence of RPBE in Q4 (mismatch ratio > 11.3) was significantly lower than that in Q1 (mismatch ratio ≤ 3.0) (*P* < 0.05). The result of multivariate logistic regression analysis showed that compared with baseline mismatch ratio <5.1, baseline mismatch ratio between 5.1 and 11.3 (OR:3.85, 95% CI 1.06–14.29, *P* = 0.040), and mismatch ratio >11.3 (OR:5.26, 95% CI 1.28–20.00, *P* = 0.021) were independent protective factors for RPBE.

**Conclusion:**

In patients with anterior circulation LVO stroke undergoing successful EVT, a large mismatch ratio at baseline is a protective factor for RPBE, which is associated with poor outcomes.

## 1. Introduction

Brain edema is a devastating complication of acute ischemic stroke, especially with large vessel occlusion (LVO); despite conservative intensive care, the mortality rate of malignant brain edema is still as high as 80% (1). Although endovascular thrombectomy (EVT) based on imaging screening has been shown to be effective and safe in patients with anterior circulation LVO stroke (2–6), approximately 45% of patients still experience poor functional outcomes after EVT (4), among whom recurrent edema is prevalent and might reduce the benefit of EVT (7, 8). Despite the limited treatments for cerebral edema, early decompressive hemicraniectomy can help reduce mortality and increase the possibility of a good functional outcome (9). Therefore, it is important to identify the risk factors for brain edema after stroke to determine the correct perioperative management.

The interaction between reperfusion and cerebral edema remains inconclusive. Experimental and clinical studies yield conflicting results. Cerebral edema deteriorates after reperfusion treatment in animal models (10–12). Nevertheless, in clinical studies, brain edema has been alleviated after recanalization (7, 8, 13). These contradictory results may indicate a complex interaction between reperfusion and edema.

A recent clinical study focused on patients with large hemispheric infarction (core volume 80–300 ml) demonstrated that when the ischemic core volume was < 130 ml, reperfusion did not affect midline shift (MLS). Conversely, when the ischemic core volume exceeded 130 ml, recanalization treatment was associated with the prevalent occurrence of MLS because of the intracranial mass effect of cerebral edema (14). Another study showed that when perfusion profiles displayed a large penumbra volume, recanalization treatment was associated with reduced brain edema, but this effect was not detected in patients with a smaller penumbra volume. Whether reperfusion therapy may reduce brain edema when the perfusion profiles showed that the ischemic core volume was minimal to moderate, depends on the penumbral volume (15). These results indicate that there seems to be a complicated correlation between perfusion status and cerebral edema, which may be influenced by a combination of factors.

There is currently limited evidence regarding the relationship between the mismatch ratio (penumbra volume/core volume) and cerebral edema in small to moderate ischemic core volume subpopulations. This study aimed to investigate the impact of the mismatch ratio at baseline computed tomography (CT) perfusion on rapidly progressing brain edema (RPBE) within 24 h after successful reperfusion in patients with anterior circulation LVO stroke.

## 2. Materials and methods

### 2.1. Patient population

**Figure 1:**
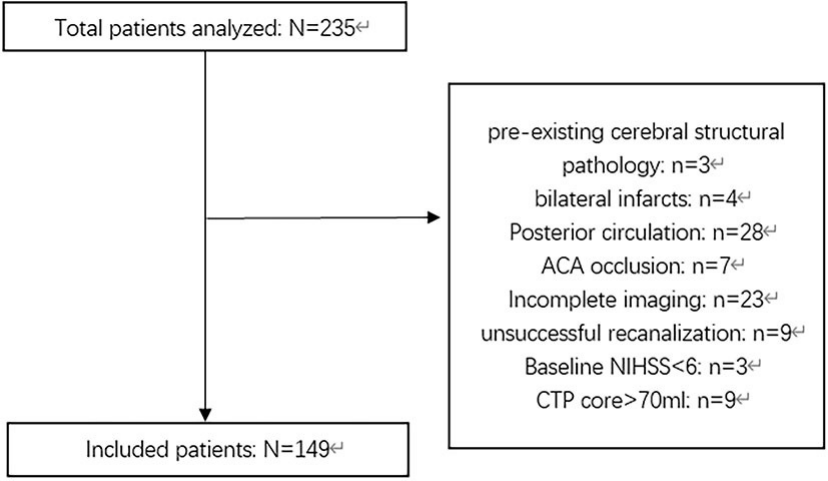
Flowchart describing the inclusion and exclusion criterion of this study. NIHSS, National Institute of Health Stroke Scale; ACA, anterior cerebral artery; OTP, Time from onset to puncture; CTP, CT-perfusion.

In this study, we retrospectively recruited patients with anterior circulation LVO stroke who underwent successful EVT at a single comprehensive stroke center (Zhejiang Provincial People's Hospital) between January 2020 and December 2021. Patients who met the following inclusion criteria were recruited: (a) age ≥ 18 years old; (b) time from stroke onset to puncture (OTP) ≤ 16 h (stroke onset is defined as the time the patient was last known to be at their neurologic baseline); (c) National Institute of Health Stroke Scale (NIHSS) score at baseline ≥ 6 and modified Rankin Scale score (mRS) before stroke < 2; (d) baseline CT angiography confirming the occlusion of the internal carotid artery (ICA) and/or proximal segment (M1 or M2) of the middle cerebral artery (MCA); and (e) for patients with OTP ≥ 6 h, baseline CT perfusion (CTP) confirming an ischemic core volume < 70 ml, and a mismatch ratio (penumbra volume/core volume) >1.8. Patients with pre-existing cerebral structural pathology, bilateral infarcts, incomplete images, known allergy to iodine, pregnancy, severe sustained hypertension (defined as systolic blood pressure >185 mmHg or diastolic blood pressure > 110 mm Hg), platelet count < 50 × 10 ^∧^ 9/L, known hereditary or acquired hemorrhagic diathesis, coagulation factor deficiency, baseline blood glucose of < 2.78 mmol/L or > 22.20 mmol, modified treatment in cerebral infarction (mTICI) score < 2b, and patients who underwent neurosurgical treatments before a 24-h CT or MR scan during the follow-up period were excluded. Figure 1 shows the inclusion and exclusion criteria used in this study.

This study was reviewed and approved by the Ethical Committee of Zhejiang Provincial People's Hospital. All patients or their legal representatives (of patients suffering from severe stroke or who were unable to speak or sign) who were suitable for reperfusion therapy were informed about the study and asked to consent for enrollment at the same time that the informed consent for treatment was provided before the reperfusion treatment. The physician informed the patients or their legal representatives that the patient's clinical and image data would be recorded for analysis and research, but no identifying information would be disclosed and no additional intervening measures would be conducted. All patients or their legal representatives signed the consent without dropping out. All the procedures were conducted in accordance with the principles of the Declaration of Helsinki. Patient data were stored confidentially in Zhejiang Provincial People's Hospital.

### 2.2. Image analysis

At baseline, whole-brain dynamic CT angiography and perfusion imaging were performed on a Toshiba Aquilion 320-slice CT scanner (Toshiba Medical Systems, Tokyo, Japan), including a non-contrast CT (NCCT) head scan (120 kV, 320 mA, contiguous 5 mm axial slices) and volume perfusion CT (VPCT) (100 mm in the z-axis, 4 s delay after start of contrast medium injection, 74.5 s total imaging duration, 80 kV, 120 mA, effective dose = 3.68 mSv, slice thickness 10 mm, collimation 32 × 1.2 mm). A total of 19 consecutive spiral acquisitions were performed. Approximately, 45 ml of iohexol (MEDRAD Stellant D SCT-212; Bayer HealthCare, Berlin, Germany) was injected at a flow rate of 5 ml/s, followed by 30 ml of saline at 4 ml/s.

We used automated commercial software (MIStar; Apollo Medical Imaging Technology, Australia) to reconstruct images and obtain ischemic core volumes, penumbra volumes, and T_max_ maps. Ischemic core volume was defined as baseline relative cerebral blood flow (rCBF) < 30% (18). The penumbra volume was defined as T_max_ > 6 s. The mismatch ratio was calculated by dividing the penumbra volume by the core volume. The collateral index was calculated by dividing the volume of delay time > 6 s by the volume of delay time > 2 s (16). The mTICI score classified the degree of reperfusion (17), and a score of 2b−3 after the EVT procedure was considered a successful recanalization (19).

**Figure 2:**
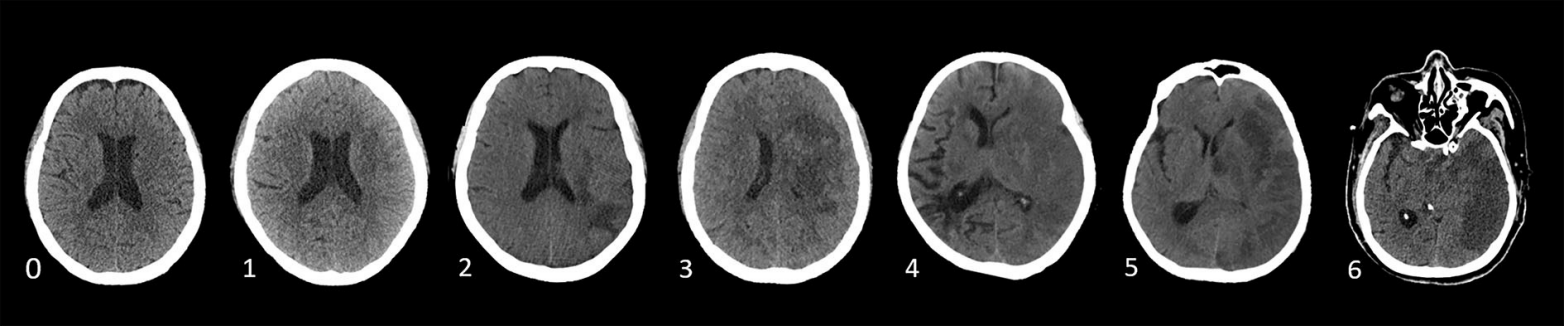
Swelling Score (0 score illustrates no swelling; a score of 1 indicates the disappearance of cortical sulci; a score of 2 implies minor effacement of the ipsilateral lateral ventricle; a score of 3 indicates the complete disappearance of the ipsilateral lateral ventricle; a score of 4 indicates the disappearance of the third ventricle; a score of 5 illustrates the shift away of the midline; and a score of 6 indicates the disappearance of basal cisterns) (20).

According to Wardlaw and Sellar (20), brain edema was assessed on a 7-point swelling scale and 0–6 points based on NCCT or MRI, which is shown in Figure 2 independently by two trained neurologists (CXY and WHY) blinded to clinical information. RPBE was defined as an increase in the swelling score by more than 2 points on follow-up NCCT or MRI performed 24 h after EVT compared with that at baseline.

### 2.3. Statistical analysis

All statistical analyses were conducted using statistical software (IBM SPSS Statistics, version 25.0). Two-sided *P* < 0.05 were regarded as statistically significant. Continuous variables were expressed as median or mean, while categorical variables were expressed as numbers with percentages. We performed a univariate logistic regression analysis to investigate the impact of the perfusion profile at baseline CTP on RPBE, including adjustments for potential confounders. Demographic, clinical, laboratory, and imaging variables associated with RPBE at a significance level of *p* < 0.05 were enrolled in the multivariate logistic regression analysis. Results were given as odds ratio (OR) with relative risks of 95% confidence interval (CI).

## 3. Results

Among 235 patients who underwent EVT, 149 were included in the study (Figure 1). The median age was 68 [interquartile range (IQR) 57.5–80.0] years, and 58.4% of the patients were male. The median values of NIHSS score (at baseline), core volume, and penumbra volume were 16.0 (IQR 12.5–20.0), 17.0 ml (IQR 7.0–43.0), and 107.0 ml (IQR 69.5–167.5), respectively. Overall, 26.2% (39/149) of patients who underwent successful recanalization experienced RPBE after EVT. Table 1 presents the baseline characteristics of the study population compared between the participants with and without RPBE. There were significant differences in the NIHSS (*Z* = 3.52, 95% CI 2.0–12.0, *P* < 0.001) and mRS scores (*Z* = 3.67, 95% CI 0.0–1.0, *P* < 0.001) at discharge between patients with and without RPBE. In addition, the occurrence of hemorrhagic transformation (χ^2^ = 22.17, 95% CI 0.29–0.59, *P* < 0.001) and mortality in hospital (χ^2^ = 9.54, 95% CI 0.06–0.36, *P* = 0.002) were significantly more prevalent in patients with RPBE (Table 2).

**Table 1:** Baseline characteristics of the study population compared between participants with and without RPBE.

**Characteristics**		**Patients without RPBE (*n* = 110)**	**Patients with RPBE (*n* = 39)**	***P-*value**
Age, ys		68 (56, 79)	70 (60, 84)	0.168
Male		65 (59.1)	22 (56.4)	0.770
Admission NIHSS		16 (12, 20)	15 (13, 19)	0.907
Admission mRS		4 (4, 5)	5 (4, 5)	0.247
SBP, mmHg		155.8 ± 28.1	154.3 ± 22.2	0.768
DBP, mmHg		90.4 ± 18.3	89.3 ± 15.4	0.732
Baseline glucose, mmol/L		7.0 (6.1, 8.7)	7.3 (6.5, 9.9)	0.380
Prothrombin time, s		11.9 (11.5, 12.8)	12.0 (11.4, 12.8)	0.841
Platelet count, 10^9^/L		177.5 (147.5, 213.0)	175.0 (117.0, 203.0)	0.260
Hemoglobin, g/L		139.0 ± 20.6	136.0 ± 14.7	0.403
Received thrombolysis		36 (32.7)	20 (51.3)	0.029^*^
Time from onset to recanalization, min		554.5 (358.0, 870.5)	393.0 (286.8, 585.8)	0.031^*^
Hypertension		49 (44.5)	20 (51.3)	0.468
Diabetes mellitus		22 (20)	8 (20.5)	0.945
History of stroke		11 (10)	4 (10.3)	0.964
Atrial fibrillation		42 (38.2)	21 (53.8)	0.097
Anticoagulant drugs		30 (27.3)	12 (30.8)	0.677
Antiplatelet drugs		13 (11.8)	8 (20.5)	0.180
TOAST classification	CE	47 (42.7)	20 (51.3)	0.290
	LAA	47 (42.7)	14 (35.9)	
	Others (mainly dissection)	10 (9.1)	1 (2.6)	
	Unknown	6 (5.5)	4 (10.3)	
Baseline brain edema scale	0	41 (37.3)	23 (59.0)	0.051
	1	45 (40.9)	9 (23.1)	
	2	24 (21.8)	7 (17.9)	
ASPECTS		8.0 (6.0, 9.0)	7.0 (6.0, 9.0)	0.219
CTP penumbra volume, ml		102.5 (68.8, 154.3)	116.0 (75.0, 186.0)	0.394
CTP core volume, ml		14.0 (6.0, 31.25)	31.0 (12.0, 65.0)	0.003^*^
CTP core volume Q1		33 (30)	5 (12.8)	0.029^*^
CTP core volume Q2		29 (26.4)	9 (23.1)	
CTP core volume Q3		27 (24.5)	9 (23.1)	
CTP core volume Q4		21 (19.1)	16 (41)	
DT + 2 s, ml		161.0 (107.8, 222.0)	167.0 (106.0, 254.0)	0.699
DT + 6 s, ml		27.5 (5.0, 59.0)	38.0 (9.0, 90.0)	0.284
Collateral index		17.5 (4.0, 31.1)	21.7 (7.8, 37.6)	0.300
Collateral index Q1		30 (27.3)	8 (20.5)	0.246
Collateral index Q2		27 (24.5)	10 (25.6)	
Collateral index Q3		30 (27.3)	7 (17.9)	
Collateral index Q4		23 (20.9)	14 (35.9)	
Mismatch ratio		5.9 (3.5, 16.6)	3.6 (1.8, 6.9)	< 0.001^*^
Mismatch ratio Q1		23 (20.9)	16 (41.0)	0.005^*^
Mismatch ratio Q2		23 (20.9)	13 (33.3)	
Mismatch ratio Q3		31 (28.2)	6 (15.4)	
Mismatch ratio Q4		33 (30.0)	4 (10.3)	

Data are demonstrated as mean ± SD, median (interquartile range), and number (percentage). The chi-Square test/Fisher test and the t-test/Mann–Whitney U-test were conducted to compare categorical and continuous variables between the RPBE and no RPBE groups as appropriate. CTP core volume (ml) in the first through fourth quartiles were ≤ 7, 7–17, 17–42, and >42, respectively; Collateral Index in the first through fourth quartiles were ≤ 4.5, 4.5–17.9, 17.9–32.4, and >32.4, respectively; mismatch ratios in the first through fourth quartiles were ≤ 3.0, 3.0–5.1, 5.1–11.3, and >11.3, respectively.

NIHSS, National Institute of Health Stroke Scale; mRS, modified Rankin scale; TOAST, Trial of Org 10,172 in Acute Stroke Treatment; CE, cardio embolism; LAA, large artery atherosclerosis; DBP, diastolic blood pressure; SBP, systolic blood pressure; ASPECTS, Alberta Stroke Program Early CT Score; CTP, computed tomography perfusion; DT, delay time.

^*^p < 0.05.

**Table 2:** The effect of RPBE on poor outcomes in hospital.

**Outcomes**	**Patients without RPBE (*n* = 110)**	**Patients with RPBE (*n* = 39)**	***Z* or χ^2^**	***P-*value**
Discharge NIHSS, median (IQR)	6.50 (2.00, 15.25)	13.00 (6.00, 40.00)	3.52	< 0.001
Discharge mRS, median (IQR)	3.50 (2.00, 4.00)	5.00 (4.00, 5.00)	3.67	< 0.001
Hemorrhagic transformation (%)	42 (38.2)	32 (82.1)	22.17	< 0.001
Mortality in hospital (%)	8 (7.3)	11 (28.2)	9.54	0.002

Data are demonstrated as median (interquartile range) or number (percentage). The chi-Square test and the Mann–Whitney U-test were conducted to compare categorical and continuous variables between the patients with RPBE and those without RPBE as appropriate.

**Figure 3:**
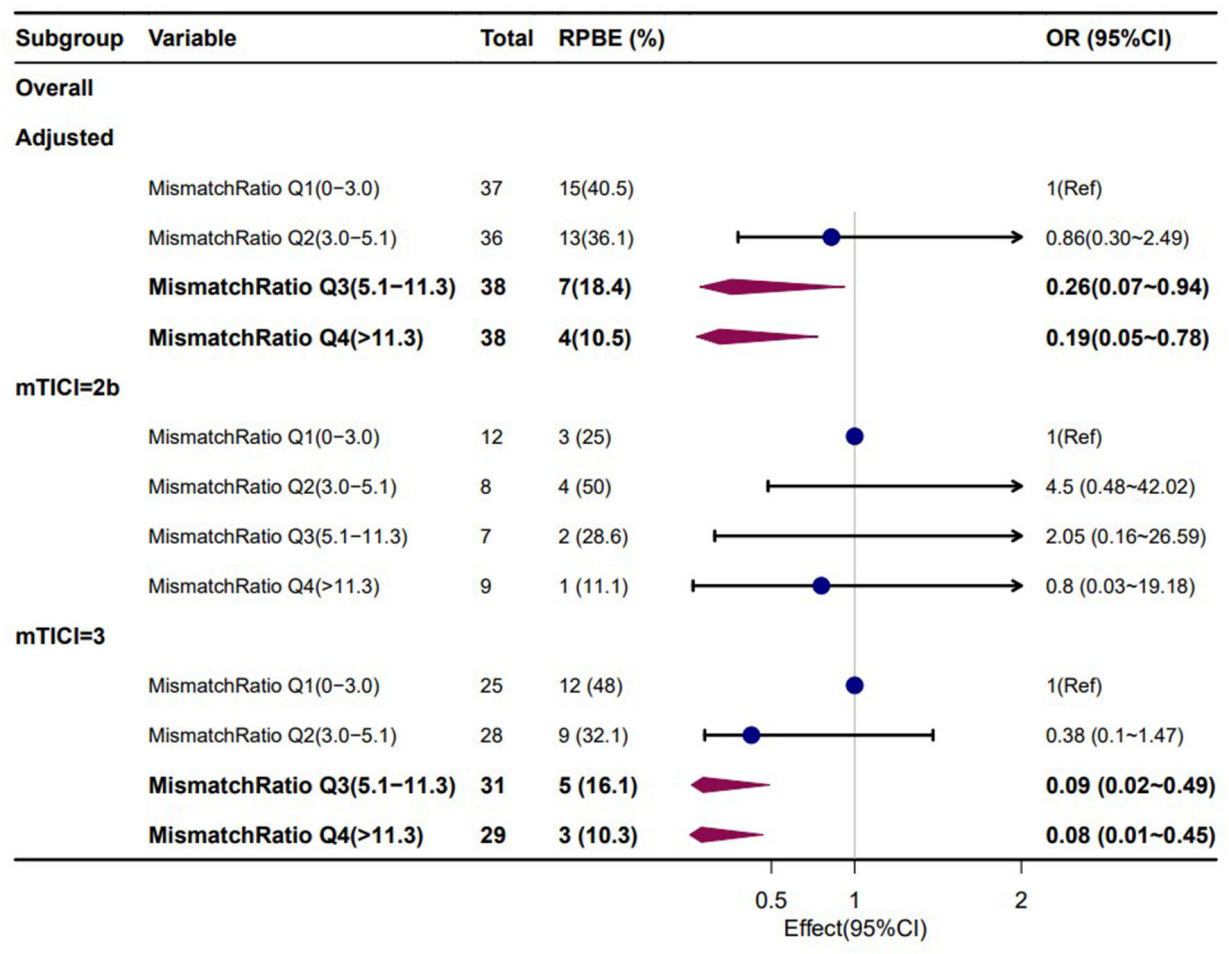
Subgroup analyses for the associations of mismatch ratio with RPBE risk, adjusted for thrombolysis and CTP core volume.

**Figure 4:**
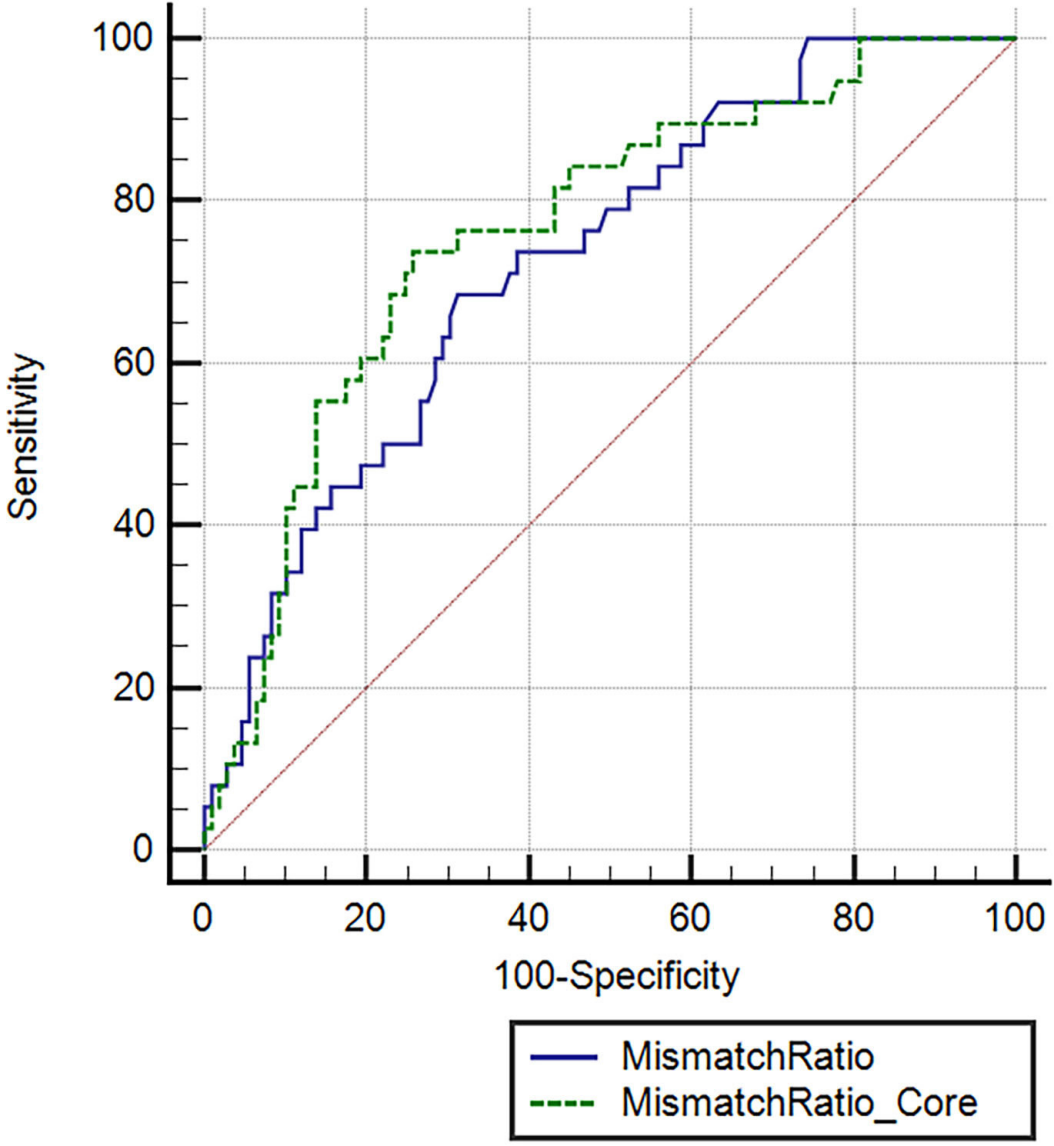
Receiver operating characteristic (ROC) curve comparing the mismatch ratio and mismatch ratio combined with ischemic core to predict RPBE. Mismatch ratio, area under the curve (AUC): 0.727 (95% CI 0.647–0.796, *P* < 0.001). Mismatch ratio combined with ischemic core, AUC: 0.763 (95% CI 0.686–0.829, *P* < 0.001).

Furthermore, a univariate logistic regression analysis was conducted to observe the correlations between demographic, clinical, and laboratory indicators; ASPECTS at baseline CT; perfusion profile at baseline CTP; and RPBE. The result showed a significant correlation among RPBE and thrombolysis (*P* = 0.031), CTP core volume (*p* = 0.019), mismatch ratio Q3 (IQR 5.1–11.3, *P* = 0.021), and mismatch ratio Q4 (IQR > 11.3, *P* = 0.005). These variables were incorporated into multivariate logistic regression analysis, which revealed that mismatch ratio Q3 (OR 0.26, 95% CI 0.07–0.94, *P* = 0.040) and mismatch ratio Q4 (OR 0.19, 95% CI 0.05–0.78, *P* = 0.021) were the independent protective factors for RPBE (Table 3). A subgroup analysis indicated that among patients with mTICI 3 scores, mismatch ratio Q3 (OR 0.09, 95% CI 0.02–0.49, *P* = 0.005) and mismatch ratio Q4 (OR 0.08, 95% CI 0.01–0.45, *P* = 0.005) were independent protective factors for RPBE (Figure 3). The area under the curve (AUC) of the receiver operator characteristics (ROC) curve was 0.727 (95% CI 0.647–0.796, *P* < 0.001). Additionally, we found that when the mismatch ratio is >5.1 and the core volume is < 42, the protective effect of RPBE was stronger than the mismatch ratio alone; and the AUC was 0.763 (95% CI 0.686–0.829, *P* < 0.001; Figure 4).

**Table 3:** Univariate and multivariate logistic regression analyses between demographic, clinical, laboratory indicators, perfusion profile at baseline CT perfusion, and RPBE.

**Variables**	**Univariate logistic regression**		**Multivariate logistic regression**	
	**Odds ratio (95% CI)**	***P*** **-value**	**Odds ratio (95% CI)**	***P*** **-value**
Received IV thrombolysis	2.28 (1.08–4.84)	0.031^*^	2.72 (1.20–6.20)	0.017^*^
CTP core volume	1.01 (1.00–1.02)	0.019^*^	1.00 (0.99–1.01)	0.644
Mismatch ratio Q3 (5.1–11.3)	0.28 (0.09–0.82)	0.021^*^	0.26 (0.07–0.94)	0.040^*^
Mismatch ratio Q4 (>11.3)	0.17 (0.05–0.59)	0.005^**^	0.19 (0.05–0.78)	0.021^*^

^**^p < 0.01;

^*^p < 0.05.

## 4. Discussion

The primary finding of our study is that RPBE after successful EVT in patients with LVO stroke is related to poor NIHSS and mRS scores at discharge, a more frequent incidence of hemorrhagic transformation, and a higher in-hospital mortality rate. Furthermore, we demonstrated that the mismatch ratio and intravenous thrombolysis were independently associated with RPBE after reperfusion therapy. Our study reveals that a large mismatch ratio is a protective factor for RPBE, which implies that a higher mismatch ratio is related to a lower possibility of RPBE. Specifically, we observed that when the mismatch ratio was >5.1 as the core volume was < 42 ml, the protective effect of RPBE was stronger than that of the mismatch ratio alone (AUC 0.763 vs. 0.727).

Many studies suggest that core volume and mismatch volume are associated with brain edema (21–23), which is consistent with the conventional understanding that serious stroke is associated with severe edema (24). Nevertheless, some of these studies did not include patients who underwent reperfusion therapy and defined brain edema by qualitative measurements, such as midline shift >5 mm or occurrence of cerebral hernia requiring hemicraniectomy. These measurements are not sensitive to mild or moderate edema; therefore, they are unsuitable for accurately monitoring a patient's condition. Identifying patients with a high risk of brain edema early is critical. This study provides a new way to assess the RPBE (an increase of more than two points in cerebral edema mass effect within 24 h). The occurrence of RPBE observed in this study (26.2%) is comparable with the 24 h net water uptake observed in previous research (20.6–22.0%) (25, 26). Although RPBE is a semi-quantitative assessment, it shows the predictive value for poor early clinical outcomes (Table 2), which is consistent with previous studies (7, 8, 27).

Considering reperfusions' effect on cerebral edema may be complicated and dependent on the perfusion profile of the ischemic tissue. Ng et al. found that recanalization was associated with reduced cerebral edema when the mismatched volume was larger than 102 ml. However, this interaction between recanalization and edema was not observed in patients with smaller mismatch volumes. A large mismatch volume indicated increased brain edema in patients who underwent unsuccessful reperfusion therapy but not in those with successful reperfusion (15). A recent clinical study focused on patients with large hemispheric infarction (core volume 80–300 ml) demonstrated that reperfusion did not affect MLS when the ischemic core volume was smaller than 130 ml. Conversely, recanalization treatment was associated with the prevalent occurrence of MLS because of the intracranial mass effect of cerebral edema when the ischemic core volume exceeded 130 ml of edema (14). These results indicate that there is a seemingly complicated correlation between perfusion status and cerebral edema, which may be influenced by a combination of factors. In contrast to previous studies, our study focused on populations with small to moderate ischemic core volume (median 17.0 ml, IQR 7.0–43.0) who underwent successful recanalization with mTICI scores of 2b to 3. Univariate analysis showed that the baseline ischemic core volume and baseline mismatch ratio were correlated with RPBE (*P* < 0.05). After dividing the mismatch ratio into quartiles and performing a chi-square test between quartiles, we found that the occurrence of RPBE in Q4 (mismatch ratio > 11.3) was significantly lower than that in Q1 (mismatch ratio ≤ 3.0) (*P* < 0.05). Multivariate logistic regression analysis revealed that mismatch ratio Q3 (OR 0.26, 95% CI 0.07–0.94, *P* = 0.040) and mismatch ratio Q4 (OR 0.19, 95% CI 0.05–0.78, *P* = 0.021) were the independent protective factors for RPBE (Table 3); the AUC-ROC curve was 0.727 (95% CI 0.647–0.796, *P* < 0.001). Furthermore, we observed that when the mismatch ratio was >5.1 and core volume was < 42 ml, the protective effect of RPBE was stronger than that of the mismatch ratio alone. The AUC was 0.763 (95% CI 0.686–0.829, *P* < 0.001; Figure 3).

The primary mechanism of cerebral edema is microvascular dysfunction and blood–brain barrier (BBB) disruption. Increased CBF volume during reperfusion is correlated with aggravation of BBB disruption (11). Approximately 26.7% of patients after endovascular treatment were found to have early BBB disruption on CT (28). Animal experiments have shown that rapidly progressing cerebral edema may occur after LVO, and increased BBB permeability was observed 20–155 min after MCA occlusion (29). A recent study reported that a larger edema volume within 24 h was associated with more edema and lesion progression (25). The mismatch ratio is the ratio of the penumbra volume to the infarct core volume, combining the infarct core with the mismatched volume. A high mismatch ratio indicates a large penumbra with a comparatively small infarct core, which is equivalent to a higher proportion of salvageable tissue. To our knowledge, this is the first study to assess early-phase edema progression with semi-quantified measurement and to reveal an interaction between RPBE with combined perfusion profiles of the ischemic core and mismatch ratio. These findings have several potential clinical implications, according to these results. Assessment of the ischemic core volume and mismatch ratio could provide prognostic information for predicting RPBE and help identify patients who are at high risk for edema and may require particular intervention.

Our study has some limitations. First, brain edema was classified into seven grades, and an increase in edema score of more than 2 points was defined as edema progression, which is a categorical variable. More accurate quantitative research is required in future. Second, the edema score is related to the degree of lateral ventricle compression; hence, patients with infarction foci close to the cortex are less likely to improve the edema score later, which may lead to bias. Third, as the implementation of mechanical thrombectomy referred to the DEFUSE 3 criteria, which required relatively small infarct cores (< 70 ml), the median core volume in our study was as small as 17.0 ml (IQR 7.0–43.0). Therefore, this may lead to selection bias; thus, the progress of brain edema after thrombectomy in patients with large infarct cores is unclear.

## 5. Conclusion

For anterior circulation LVO stroke patients with successful EVT, RPBE is associated with poor outcomes. A large mismatch ratio at baseline is a protective factor for RPBE in patients with mild-to-moderate core volume. Furthermore, these results could help identify patients with a high risk of edema and who probably require appropriate intervention.

## Data availability statement

The raw data supporting the conclusions of this article will be made available by the authors, without undue reservation.

## Ethics statement

The studies involving human participants were reviewed and approved by the Ethical Committee of Zhejiang Provincial Peoples' Hospital. The patients/participants provided their written informed consent to participate in this study.

## Author contributions

YShao and YG designed the study. XC, HW, JX, and YShan collected the data. YShao, XC, and JZ performed the statistical analysis, interpreted the results, and drafted the article. YShao, XC, YG, and PW critically revised the article. All authors contributed to the article and approved the submitted version.
